# Influence of Reinforcing Agents on the Mechanical Properties of Denture Base Resin: A Systematic Review

**DOI:** 10.3390/polym13183083

**Published:** 2021-09-13

**Authors:** Alhanoof Aldegheishem, Modhi AlDeeb, Khold Al-Ahdal, Mohammad Helmi, Eman I. Alsagob

**Affiliations:** 1 Clinical Dental Sciences Department, College of Dentistry, Princess Nourah Bint Abdulrahman University, Riyadh 11671, Saudi Arabia; asaldegheishem@pnu.edu.sa; 2 Department of Prosthodontic Dental Science, College of Dentistry, King Saud University, Riyadh 11451, Saudi Arabia; maldeeb@ksu.edu.sa; 3 Department of Restorative Dentistry, College of Dentistry, King Saud University, Riyadh 11451, Saudi Arabia; kalahdal@ksu.edu.sa; 4 Periodontics and Community Dentistry Department, College of Dentistry, King Saud University, Riyadh 11451, Saudi Arabia; mhelmi@ksu.edu.sa; 5 Preventive Dental Sciences Department, College of Dentistry, Princess Nourah bint Abdulrahman University, Riyadh 11671, Saudi Arabia

**Keywords:** denture polymer, filler, polymethyl methacrylate (PMMA), properties

## Abstract

Knowledge about the influence of fillers in denture base resin is vague. This systematic review aimed to report the reinforcing effect of fillers on the mechanical properties of denture base resin by following PRISMA guidelines. Two electronic databases (Pubmed/Medline & Web of Science) were searched for articles using the keywords: fibers in denture base, fillers in denture base, and reinforcement of denture base. Laboratory studies complying with the inclusion criteria were reviewed according to the set protocol. The established focus question was: “Do reinforcing fillers positively influence the mechanical properties of polymethyl methacrylate (PMMA) heat polymerized denture base material?” A total of twenty-nine relevant papers qualified for final inclusion. Of these, 24 were determined to have a moderate risk of bias. Micron or nano-sized metal/metal oxides particles and glass fibers were the frequently used reinforcing agents. The trend of evaluating fractural strength (FS) was common. Most of the studies limited the use of reinforcing agents up to 5 wt.%. FS, fracture toughness (FT), and impact strength (IS) tend to increase if the fillers are chemically bonded and well-dispersed in denture base resin. Though fillers with a higher elastic modulus increase the hardness of the reinforced denture base resin, they compromise other mechanical properties. Well-dispersed lower filler loading PMMA denture base resin can enhance the FS, FT, and other related mechanical properties.

## 1. Introduction

Vulcanite, bakelite, celluloid, and phenol formaldehyde were the materials that were the most commonly used for denture base resin fabrication before the advent of polymethyl methacrylate (PMMA) [1]. However, these materials had associated disadvantages such as poor aesthetics, high dentistry, brittleness, difficult manipulation, being prone to staining, etc. [2,3].

PMMA has been a widely used denture base resin for decades [4,5,6,7]. This material was reported for the first time by Redtenbacher in 1843 [1]. However, the use of PMMA as a denture base resin started in the 1930s. This material possesses many advantages such as low cost, ease in fabrication, polishing, biocompatibility, satisfactory aesthetics, low density, and colour matching ability [1,8,9]. In contrast, the associated disadvantages, which include insufficient hardness, flexural strength, toughness, and elastic modulus, make this material highly prone to fracture and cause clinical failure [10,11,12,13].

The fracture of dentures is a common clinical problem and generally results from two different kinds of forces, namely flexural fatigue and impact [14,15]. Due to repeated flexing and loading of a denture base resin, flexural fatigue occurs [16]. The development of microcracks in the region of stress concentration and the fusion of these cracks leads to ever-growing fissures, causing structural failure [17,18]. Impact failures usually occur outside of the mouth because of a sudden blow to the denture or accidental dropping whilst cleaning, coughing, or sneezing [15].

Over the years, many investigators have researched how to alter the polymeric structure of denture base resin. However, experimental work was ceased for one reason or another [19]. Recently, investigators have focused on reinforcing the denture base resin by incorporating fillers of different shapes, sizes, forms, and orientations [20]. With the advent of nanotechnology, nanofillers are increasingly being used to boost the mechanical properties of denture base resin [21,22]. Nanofillers that are oxides, metals, glass, cellulose, and polymers are available as additives with the potential of altering the mechanical properties of PMMA-based denture base resin [1,23,24]. The reinforcement effects of these micron or nano-sized particles have been reported as being instrumental in boosting the properties of denture base resin [21,25]. However, there is a lack of knowledge and understanding regarding the effectiveness of these fillers and their optimal loading in denture base resin. Therefore, this systematic review aimed to assess, compare, and explore the effects of reinforcing agents on the mechanical properties of heat-cured PMMA denture base resin.

## 2. Material and Methods

### 2.1. Focus Questions

The focus question of this study was “Do reinforcing fillers positively influence the mechanical properties of heat polymerized PMMA denture base resin?”.

### 2.2. Search Strategy

The Medline/PubMed and Web of Science databases were last searched on April 21 2021. Only dental and materials science journals were explored, and the data were composed for further perusal. The keywords employed for the search strategy are reported in Table 1.

### 2.3. Eligibility Criteria

The published studies with a sufficient sample size and the statistically analyzed results were included. The published studies had to have been a laboratory study with purely mechanical outcomes, i.e., flexural strength (FS), elastic modulus (EM), impact strength (IS), flexural modulus (FM), tensile strength (TS), compressive strength (CS), surface hardness (SH), and fracture toughness (FT).

### 2.4. Inclusion and Exclusion Criteria

Only the laboratory studies that aimed to evaluate the reinforcement effect of filler on the mechanical properties of conventional PMMA-based heat polymerized denture base resin were included.

Excluded were in vivo, clinical trials, the reinforcement of a denture base resin other than PMMA, denture repair, and fixed prosthesis or overdenture related studies. Review articles, meta analyses, letters to the editor, case report/series, literature reviews, commentaries, incomplete studies, and articles published in a language other than English were also excluded.

### 2.5. Risk of Bias

The methodological quality of each included study was independently evaluated by the two reviewers, as adapted and adjusted from another systematic review of in vitro studies [26] to achieve the specified goal. The criteria used to assess the risk of bias was based on the mentioning of a sample fabrication technique, sample size, sample allocation or concealment, sample power calculation, blinding of the operator, ISO/ADA standards, and outcome reported. If the criteria written in the study were clear, it received a score of “0”. If the required data were vague or uncertain, the score was set as “1”, and if a specific approach was undisclosed, the score was established as “2”. Articles that secured a score of 0 to 4 were determined to have a low risk of bias; counts between 5 to 9, were consider to be at a moderate risk of bias; and counts between 10 to 14 were considered to have a high-risk of bias.

## 3. Results

### 3.1. Data Selection

**Figure 1:**
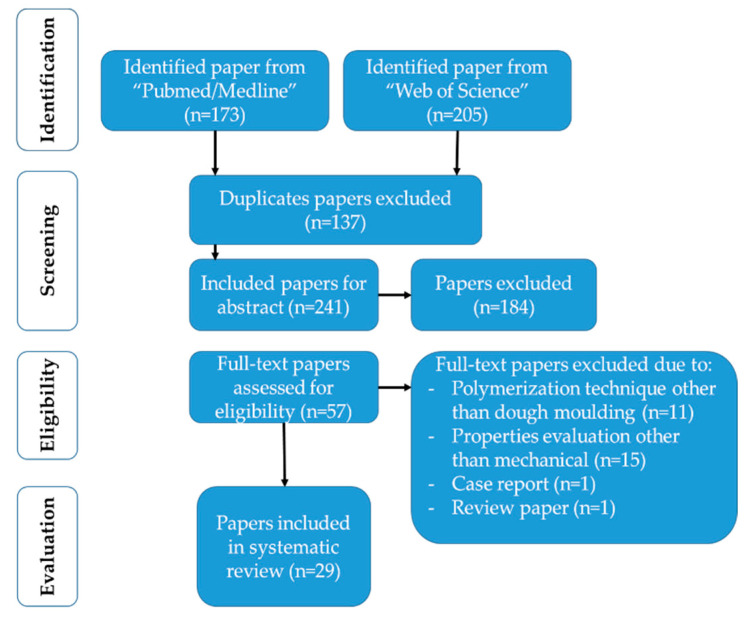
Flowchart of the systematic review.

A total of 378 potentially relevant papers were retrieved due to the primary search conducted from 1 January 2010 until 21 April 2021. The data were imported into Endnote X9 software (Thompson Reuters, Philadelphia, PA, USA) to remove duplicates (137 papers). Consequently, 241 papers were included for the review of their abstracts. After careful abstract perusal by two independent reviewers (A.A. and E.I.A.), 82 papers were excluded due to research on implant/finite element analysis/fixed prosthesis; 35 papers were excluded due to biological/clinical/review studies; 39 papers were excluded due to research on denture repair/soft liner/denture teeth/framework; and 28 papers were excluded due to research on a new polymer or polymerization technique. The remaining 57 titles were thoroughly assessed by two pairs of independent reviewers (M.A. and M.H.; A.A. and K.A.). A further 28 were eliminated based on the evaluation of denture base resin properties other than the mechanical ones. Finally, 29 papers were selected and included that fulfilled the criteria according to the preferred reporting items for systematic reviews and meta analyses (PRISMA) statement (Table 2, Figure 1) [27].

### 3.2. Quality Assessment

Table 3 represents the risk of bias outcome of the included studies. Out of 29 studies, 4 presented a high risk of bias, whereas the majority of the included studies (i.e., 24) showed a moderate level of bias. Only one study showed a low risk of bias. The trend of sample allocation or concealment was observed to be uncommon among the investigators. Only one study mentioned it. Moreover, the use of the the sample power calculation before testing also seems to be sporadic in laboratory studies. Surprisingly, none of the included studies reported the “blinding of the operator”. The details are described in Table 3.

### 3.3. Data Analysis

The outcome of this systematic review generated 29 studies. Of these, the majority of them evaluated metal or metal oxides as reinforcing agents [29,30,33,36,42,46,48,49,50,55,56], either in micron or nano-sized particles. Ceramic oxide particles were also equally interesting as reinforcing agents for the investigators [28,29,30,32,33,35,44,45,47]. All of these studies used nano-sized particles, except for Al-Bakri et al. [35], in which 1.5 µm sized glass fillers were used. Glass fibers and other types of polymeric fibers were of also interest to investigators [31,32,34,37,38,39,40,41,51,52]. There was less of an inclination towards the use of minerals such as mica, borax, boric acid, and colemanite as reinforcing agents in denture base resin [43,53]. The use of treated reinforcing agents was also found in most of the included studies.

The trend of evaluating the fractural strength (FS) and fracture toughness (FT) were the common testing parameters. However, the investigators were also interested to see the reinforcing effect of fillers on surface hardness (SH) [30,43,46,47,53,54,55,56]. On the contrary, the IS of the reinforced denture base resin was evaluated in very few studies [45,48,53]. While the compressive strength [50] and the fracture resistance [40], each as testing parameters, were used in a single study.

As observed in Table 4, most of the studies limited the addition of reinforcing agents up to 5 wt.% of the denture resin base polymer. All of these studies demonstrated enhanced mechanical properties. However, a study by Cevik and Yildirim-Biceret [32] showed decreased FS using 0.1 to 5.0 wt.% of filler loading. In contrast, a study by Yu et al. [38] showed increased FS and FT at 5.3 wt.% and 7.9 wt.% loadings of GL/UHMWPE fibers. Additionally, a study by Mathew et al. [52] demonstrated statistically higher FS at 10 wt.% loadings of hydrogen plasma-treated polypropylene fibers. The other studies either claimed no effect on FS [29] or reported enhanced FM, SH, or CS at elevated filler loading levels [43,44,50].

## 4. Discussion

In many clinical scenarios, a complete denture prosthesis is still a viable option for many reasons. Throughout the years, a wide range of micron or nano-sized fillers has been used to enhance the mechanical properties of denture base resin. This in-depth systematic review primarily focused on evaluating the reinforcing agents and their effect on the mechanical properties of denture base resin. At the time of this systematic review, several other authors have also reviewed the literature on this subject matter [9,10,57,58,59]. However, none of them reviewed the topic systematically, or if it was done systematically, it was limited to the effect of TiO_2_ nanoparticles only [60]. For the explicitness and clarity of the results, every single reviewed study was presented in Table 4 that described the testing method, the reinforcing agent used, and the outcome reached.

Regarding the quality of the included laboratory studies, it seems that most of the studies did not emphasize the risk of bias assessment. It is important to mention that the risk of bias assessment is a vital mechanism for any research design and to establish transparency and reproducibility of findings. The majority of the studies showed confounding and measurement biases by testing the samples without blinding the operator. The assessment of the methodologic quality of a laboratory study relies on the transparency of the study design, study conduct, sample size, sample allocation/concealment, sample power calculation, and if the materials testing standards were followed.

The statistically higher FS and FT using silanized nano organoclay fillers in a study by Shakeri et al. [28] might suggest that using 3-trimethoxysilylpropyl methacrylate (MPS) containing methoxy groups, which upon hydrolysis, forms a silanol group and reacts with the OH group on clay platelet, while the vinyl group participates in a polymerization reaction with the PMMA, which is helpful in increased FS and FT. Hence, there is a relatively strong bond formation at the nanoparticle–matrix interface. We assume that the selection of silane is significant for the linkage of a particular reinforcing agent with PMMA resin. This might be the reason that the study by Kul et al. [29] showed indifferent mechanical properties. Homogenous distribution of reinforcing agents with high modulus, high surface area, and stiffness is crucial for enhanced FS and FT, as observed in a study by Rahaman et al. [31]. Moreover, shape, size, distribution/orientation of filler/fiber in a resin matrix, and connection to the resin matrix are imperative factors, as perceived by Karci et al. [33]. This proposition was confirmed in a study by Uyar et al. [34], who demonstrated that aligned nanofibers could improve the FS at lower filler loading compared to non-aligned nanofibers. Furthermore, unidirectional aramid fibers were more effective than woven, non-woven, and paper types in reinforcing the FS and modulus of denture base resin as witnessed by Yu et al. [37]. In contrast, studies by Cevik et al. [32] and Al-Bakri et al. [35] demonstrated reduced FS at lower filler loading. This might suggest that selection of silane primer is crucial in the final properties of the composite [61,62].

In another study by Yu et al. [38], the combined reinforcing effect of fibers was evaluated and was found to be supportive in the enhanced FS of the filler-PMMA composite. If the selected reinforcing agent has a higher modulus of elasticity, it endures stresses without deformation, resulting in increased FS [39,41,63], irrespective of whether the reinforcing agent is treated or not [42]. However, decreased FS might be observed due to the poor orientation of the reinforcing agent [43]. It is desired that the reinforcing agent be oriented along the plane of the denture plate for optimal FS. In addition, adequately silanized nanofillers could form a covalent bond between the nanofillers and the PMMA matrix at lower filler loading [64]. However, at elevated filler loading, the interparticle spacing is lessened, permitting increased agglomeration [44,45,46,49]. When the reinforcing agent’s content surpassed the optimum amount, agglomeration occurs. At agglomerated particles, stress is generated, which lowers the mechanical properties of composite material [65].

Although the reinforcing agents might possess outstanding mechanical properties, they could be unsatisfactory for the reinforcement of denture base resin. Such is the case for nylon, as experimented upon by Galav et al. [51], suggesting that high molding shrinkage leads to warpage and high water absorption, making nylon fibers unsuitable for reinforcing the denture base resin. Similarly, fiber length and its adhesion with the resin matrix are crucial for the flexural properties of composite material [52]. Nonetheless, reinforcing agents in denture base resin improves the FS, however, the efficacy hinges on several factors, including the material type, form, % of loading, surface treatment, and orientation of the reinforcing agent [53,54,55,56].

The toughness of reinforced denture base resin largely rests on how well the interfacial adhesion is between a reinforcing agent and a denture base resin [40,66]. PMMA denture base resin is a brittle material. Untreated particles become a source of void formation at the interfacial area in the absence of a coupling agent. Nanoparticles have the affinity to agglomerate due to van der Waals forces and high interfacial tension; it is therefore likely that detachment between the two phases occurs. However, if the reinforcing particles are small, they can fit in the interstitial of polymer particles to produce a heterogeneous mixture and will not easily force the displacement of the segments of the polymer chain during the applied load [67]. Moreover, crack growth is interrupted by the particle/fiber, which can absorb some energy before facilitating further crack growth. The included studies validated these findings [28,34,36,37,41,47,48]. However, although toughness is an important testing parameter, it is a preliminary testing method and is usually employed when the performance of the material is unknown. On the contrary, tests such as fatigue resistance are dynamic and depict oral conditions in a true sense. However, we noticed a single study by Gurbuz et al., who opted for the fatigue resistance evaluation of reinforced denture base resin [40].

The agglomeration of reinforcing agents increases the hardness of a material [68]. This might be because of the formation of a thick immobilized PMMA layer that resists indentation around the reinforcing agent [30]. Well-dispersed fillers help in maintaining the strength of reinforced denture base resin [56]. Low filler loading might homogenously distribute within the denture base resin, fill the void/space of the inter-polymeric chain, and limit their movement. Similar findings were suggested by Gad et al. [30], Balos et al. [47], Demir et al. [53], Zhang et al. [55], and Naji et al. [56]. However, decreased microhardness at elevated loading might suggest that the resin is not adequately reinforced or that the EM of a reinforcing agent is lower than that of the resin itself. Hence, lower hardness values at an elevated filler loading were observed. However, in the case of lower hardness at 0.6 wt.% and 0.9 wt.% for halloysite nanotubes [54], this might be attributed to lower density, i.e., 2.14–2.59 g/cm^3^ [69] or poor adhesion to the polymer at higher loading.

The evaluation of an IS is a vital parameter for denture base resin. In a study by Al-Harbi et al. [45], we observed justified results that the addition of nanodiamond filler decreases the IS of a denture base resin. The decreased IS might suggest the agglomeration or loosely attached cluster formation of these nanofillers. However, statistically higher IS in a study by Asar et al. [48] advocates that hard metal oxide micron-sized filler (i.e., 8.6 µm–12.4 µm in range) might act as space/void filler between the average size (121.2 µm) of a PMMA polymer bead and hence increase the crack length during the fracture. Increasing crack length can result in an increase in energy absorption before fracture and can improve the IS. In contrast, the particle used in Demir et al. [53] was a 45 µm colemanite-sized filler, which showed decreased IS at 2 wt.% and 3 wt.% loadings. This could be why agglomeration and loosely attached clusters may form at higher loadings. Increased IS at 1 wt.% might promote a synergistic effect.

It is difficult to assess the usefulness of organoclay particles investigated by [28] or the halloysite nanotubes as investigated by [54]. The comparison of the results with other reinforcing agents is difficult based on just one or two studies. However, it seems that clay particles can enhance the mechanical properties of denture base resin if the appropriate weight fractions of the said particles are used. A 0.25 wt. % to 0.5 wt. % of clay particles could be recommended for enhancement of mechanical properties. However, further laboratory studies related to the use of clay particles are necessary to comprehend and understand this phenomena.

Another important development in the science of reinforcing the material is the use of hybrid fillers. It is a fascinating technique that combines the individual properties of reinforcing agents in denture base resin. Yu et al. observed that the hybrid effect of GL/PE is instrumental in enhancing the FT and FS [38]. Similarly, a combination of ZrO_2_-ABW nanoparticles helps to raise the FS and SH by 52% and 27%, respectively, when incorporated in denture base resin in a laboratory study by Zhang et al. [55].

The findings of this systematic review suggest that though the reinforcing agents improve the FS, FT, IS and hardness, the effect of the reinforcing agents varies in terms of several factors such as reinforcing the agent’s shape, size, form, orientation, concentration, and surface treatment. The hybridization of fillers in denture base resin is another approach for the enhancement of the mechanical properties. A combination of fillers of different sizes may produce a synergistic effect. The potential advantages of filler reinforced composites largely rely on the filler content and its dispersion and surface nature. Based on laboratory studies, the clinical efficacy of reinforcing materials cannot be established because of certain clinical factors, such as the presence of a moist environment or parafunctional habits that may negatively affect the reinforcing effect. Moreover, heterogeneity of the study design, technique, followed protocols, operator handling, and the environment where tests are performed can influence the findings. Hence, clinical trials are necessary to predict the reinforcement effect.

There is no established guideline available for evaluating and rating the methodological quality of a laboratory study with an associated risk of bias. Therefore, the risk of bias tool was adapted from different published papers and was tailor modified for use in the present study. Furthermore, this review was restricted to heat-cured PMMA denture base resin only and did not account for any other denture base resin or polymerization techniques other than compression moulding technique. Moreover, this systematic review was restricted to paper in the English language, and unpublished data or conference proceedings were not included in this review. Consequently, publication bias is probable. Certain recommendations are necessary for future research and reviews: (1) the investigators must provide detailed information related to reinforcing the agent’s size and shape. (2) The sample power calculation needs to be performed before testing and detecting a hypothesized effect size. (3) There should be strict ISO testing standards for the investigators to follow. (4) Other databases such as “Scopus” and “Cochrane” should be included in future reviews to broaden the search for a wider view on the subject.

## 5. Conclusions

Considering the limitations and the diversified findings of this systematic review, the following conclusions were drawn:

(1) A wide variety of denture base resin reinforcing agents is available, which makes it difficult to compare results;
(2) FS is the most commonly used testing method among investigators for the evaluation of the reinforcing effect on denture base resin;
(3) Up to 5 wt.% of filler loading in denture base resin seems practicable and effective in reinforcing the denture base. At higher filler loadings, the FS of a denture base resin is reduced;
(4) The FT of a denture base resin increases provided that there is an interfacial adhesion between the reinforcing agent and a denture base resin;
(5) The agglomeration of the reinforcing agents increases the SH of a material. Decreased microhardness at elevated loadings might suggest that the denture base resin is not reinforced adequately or the EM of a reinforcing agent is lower than that of the resin itself;
(6) Agglomeration or a loosely attached reinforcing agent in a resin matrix decreases the IS;
(7) The hybridization of fillers in denture base resin seems to be a viable option.

## Figures and Tables

**Table 1:** Search strategy.

Source	Criteria
Database	Medline/Pubmed, Web of Science
Date of publication	1 January 2010–21 April 2021
Keywords	fibers in denture base fillers in denture base reinforcement of denture base
Language	English
Type of paper	Laboratory research
Inclusion criteria	Laboratory studies that evaluated the mechanical properties of PMMA heat polymerized denture base resin
Exclusion criteria	Review, meta analysis, case report/series, clinical trial, denture repair, overdenture, denture teeth, implant/finite element analysis/fixed prosthesis, new polymer or polymerization technique
Journal category	Dental, medline, materials science

**Table 2:** The outcomes of the screening events.

Keywords	Database Source			
	Pubmed/Medline		Web of Science	
	Retrieved	Selected	Retrieved	Selected
Fibers in denture base	525	9	97	3
Fillers in denture base	22	9	29	3
Reinforcement of denture base	99	7	79	

**Table 3:** Risk of bias tool (adapted and modified from [26]).

Ref	Sample Fabrication Technique	Sample Size	Sample Allocation or Concealment	Sample Power Calculation	Materials Testing Standards		Blinding of Operator	Objective/Finding Mentioned	Risk of Bias
[28]	1	0	2	2	0		2	0	Moderate
[29]	0	0	2	2	0		2	0	Moderate
[30]	1	0	2	2	2		2	0	High
[31]	0	0	2	2	0		2	0	Moderate
[32]	0	0	2	2	0		2	0	Moderate
[33]	0	0	2	0	0		2	0	Low
[34]	1	1	2	2	2		2	0	High
[35]	1	0	2	2	0		2	0	Moderate
[36]	1	0	2	2	2		2	0	Moderate
[37]	0	0	2	2	0		2	0	Moderate
[38]	0	0	2	2	0		2	0	Moderate
[39]	0	0	1	2	2		2	0	Moderate
[40]	0	0	2	2	0		2	0	Moderate
[41]	0	0	2	2	0		2	0	Moderate
[42]	1	2	2	2	2		2	0	High
[43]	0	0	2	0	2		2	0	Moderate
[44]	1	0	2	2	0		2	0	Moderate
[45]	0	0	2	2	0		2	0	Moderate
[46]	1	0	0	2	0		2	0	Moderate
[47]	1	1	2	2	2		2	0	High
[48]	0	0	2	2	0		2	0	Moderate
[49]	1	0	2	2	2		2	0	Moderate
[50]	1	0	2	2	0		2	0	Moderate
[51]	0	0	2	2	0		2	0	Moderate
[52]	0	0	2	2	2		2	0	Moderate
[53]	0	0	2	2	0		2	0	Moderate
[54]	0	0	2	2	2		2	0	Moderate
[55]	0	0	2	2	0		2	0	Moderate
[56]	0	0	2	2	0		2	0	Moderate

**Table 4:** Included studies with types of reinforcing agents used in PMMA heat-cured denture base resin and their outcomes corresponding to testing parameters.

Ref.	Testing Method	Reinforcing Agent/s Used	Outcome
[28]	FS, FT	Treated 0.25 and 0.5 wt.% double-modified organoclay nanoparticles	↑↑ FS and FT in both 0.25 and 0.5 wt.% nanoparticles groups
[29]	FS	Ag, TiO_2_, ZrO_2_, Al_2_O_3_, SiC, SiC-nano, Si_3_N_4_, and HA-nano in ratios of 10 wt.% to PMMA	↔ between the study groups
[30]	SH	ZrO_2_ (14 nm), SiO_2_ (12 nm), and diamond nanoparticles (19 nm) in concentrations of 0%, 0.5%, 1.0%, 2.5%, and 5.0% by weight of acrylic powder	↑↑ in SH compared to Control
[31]	FS, FM	Microcrystalline cellulose with 2 or 5% by weight	↑↑ FS, FM in 5 wt.% group
[32]	FS	1 wt.% or 5 wt.% of SiO_2_ or prepolymer nanoparticles	↓↓ FS in experimental groups compared to control group
[33]	FS	1, 3, or 5 wt.% of Al_2_O_3_ (18 nm), SiO_2_ (15 nm), or TiO_2_ (13 nm) nanoparticles	1 wt.% of nanoparticles ↑↑ FS
[34]	FS, EM, FT	Polyvinyl alcohol aligned and non- aligned nanofiber with 0.05% w/w, 0.25% w/w, 1% w/w, or 1.25% w/w.	Aligned nanofiber ↑↑ increased the mechanical properties of denture base resin
[35]	FS	Treated glass fillers (1%, 2.5%, 5%, and 10% by weight) 1.5 µm sized	FS ↓ as glass filler uploading ↑
[36]	FT	Silanated nano barium titanate at 5 wt.%	↑↑ FT in the experimental group
[37]	FS, FM	Treated aramid fibers with four orientations (unidirectional, woven, non-woven, and paper-type)	unidirectional and woven aramid fibers ↑↑ FS & FM
[38]	FS, FM, FT	GL, aramid, and UHMWPE fibers at volume concentrations of 2.6%, 5.3%, and 7.9%, respectively	Combination of GL/UHMWPE fibers showed ↑↑ FT and FS
[39]	FS	GF, aramid, nylon at 4 wt.% (5 mm in length)	↑↑ FS in GF and aramid reinforced groups
[40]	FR	GF (chopped strand mat, continuous or woven) at 2.5, 3, 4, 5 vol.%	↑ fracture resistance in all forms of GF
[41]	FS, FT, FM	E-Glass FiBER FORCE	↑ mechanical properties were observed
[42]	FS	Treated and untreated ZrO_2_ nanotubes (8 µm in length)	2.0 wt.% ZrO_2_ nanotubes ↑ FS
[43]	FS, SH	Silane treated fine or coarse mica particles (30 µm and 131 µm) at 10 vol.% or 20 vol.%	↓ FS, however, ↑ SH with 20 vol.% mica reinforcement
[44]	FS, FM	Silanized nano SiO_2_ (36 nm in size) at 0.25, 0.5, 1, 5, 10, and 15 wt.%	1 wt.% presented ↑ FS while 10 % 15 wt.% showed ↑ FM
[45]	FS, IS	Nanodiamond (30–40 nm in size) at 0.5, 1, and 1.5 wt.%	0.5 wt.% reinforced PMMA displayed ↑↑ FS. Control group showed ↑↑ IS
[46]	FS, SH	1, 2, 3, 4, 5, 10, 15, or 20 wt.% aluminum borate whiskers (5–30 µm in length)	Silanized ABWs ↑ FS, SH. Optimal loading was 5 wt. % while 15 wt.% for SH
[47]	SH, FT	0.023%, 0.046%, 0.091%, 0.23%, 0.46%, and 0.91% by vol. of SiO_2_ nanoparticles	0.023% resulted in ↑ SH and FT
[48]	IS, FT	1% TiO_2_ and 1% ZrO_2_, 2% Al_2_O_3_, 2% TiO_2_, and 2% ZrO_2_ by volume	IS and FT values ↑↑
[49]	FS	Silanized Al_2_O_3_ (0.1, 0.2, or 0.4 wt.%) 18–23 µm in size	0.1 wt.% Al_2_O_3_ showed ↑↑ FS
[50]	TS, FS, CS	10%, 20%, and 30% by volume Ag and Al	CS ↑↑ while TS and FS ↓ at 30 vol.%
[51]	FS	Treated S-glass fiber, nylon fiber	↑↑ in S-glass reinforced PMMA
[52]	FS	Hydrogen plasma-treated polypropylene fibers (2.5. 5 & 10 wt.%)	↑↑ FS in tested groups
[53]	FS, IS, SH	Borax, boric acid, colemanite	The addition of 1% Colemanite to PMMA ↑ mechanical properties
[54]	FS, EM, SH	Halloysite nanotubes at 0.3, 0.6, and 0.9 wt.%	0.3 wt% halloysite nanotubes ↑ mechanical properties
[55]	FS, SH	Silanized nano ZrO₂ and nano aluminum borate whiskers at 1, 2, 3, and 4 wt.%	The mechanical behaviours of silanized ZrO₂-ABW/PMMA composites ↑↑ improved
[56]	FS, SH	Treated 2.5 wt%, and 5 wt% of TiO_2_ nanotubes	↑↑ FS and SH in experimental groups

Key: ↑↑ = significant increase, ↑= increase, ↔ = no significant change, ↓ = decrease, ↓↓ = significant decrease, FRC = fiber reinforced composite, GL = glass, GF = glass fiber, UHMWPE = ultra high molecular weight polyethylene, FR = fatigue resistance, FS = flexural strength, SH = surface hardness EM = elastic modulus, IS = impact strength, FT = fracture toughness, FM = flexural modulus, TS = tensile strength, CS = compressive strength.

## Data Availability

Not applicable.
